# The ADF/Cofilin-Pathway and Actin Dynamics in Podocyte Injury

**DOI:** 10.1155/2012/320531

**Published:** 2011-11-30

**Authors:** Beina Teng, Alexander Lukasz, Mario Schiffer

**Affiliations:** Division of Nephrology, Department of Medicine, Medical School Hannover, 30625 Hannover, Germany

## Abstract

ADF/cofilins are the major regulators of actin dynamics in mammalian cells. The activation of ADF/cofilins is controlled by a variety of regulatory mechanisms. Dysregulation of ADF/cofilin may result in loss of a precisely organized actin cytoskeletal architecture and can reduce podocyte migration and motility. Recent studies suggest that cofilin-1 can be regulated through several extracellular signals and slit diaphragm proteins. Cofilin knockdown and knockout animal models show dysfunction of glomerular barrier and filtration with foot process effacement and loss of secondary foot processes. This indicates that cofilin-1 is necessary for modulating actin dynamics in podocytes. Podocyte alterations in actin architecture may initiate or aid the progression of a large variety of glomerular diseases, and cofilin activity is required for reorganization of an intact filtration barrier. Since almost all proteinuric diseases result from a similar phenotype with effacement of the foot processes, we propose that cofilin-1 is at the centre stage of the development of proteinuria and thus may be an attractive drug target for antiproteinuric treatment strategies.

## 1. Introduction

Glomerular visceral epithelial cells (podocytes) play a central role in maintenance of the Glomerular Filtration barrier by preventing the loss of high-molecular-weight molecules. The podocyte is a highly specialized and polarized cell type that consists of three parts: the cell body, the primary foot processes, and the secondary foot processes. The interdigitating foot processes completely cover the outer surface of the glomerular capillary and form a filtration slit that is spanned by a membranelike structure; this is called the slit diaphragm [1]. Actin filaments are the structural backbone component of podocyte foot processes. Protein complexes of slit diaphragm that regulate or stabilize the actin cytoskeleton are therefore essential for the maintenance of an intact glomerular filtration barrier [2]. When podocytes are injured, they undergo dramatic actin cytoskeletal changes. These cytoskeletal changes lead to retraction of secondary processes and loss of functional filtration slits; this is termed foot process effacement. Foot process effacement is a dynamic and reversible process that contributes to the development of massive proteinuria in human glomerular diseases [3]. 

Actin is one of the most abundant and highly conserved proteins in many eukaryotic cells. It is involved in many different cellular processes that are essential for cell growth, differentiation, division, membrane organization, and motility [4]. The dynamics of actin filaments (F-actin) assembly/disassembly and organization in cells are regulated by several actin-binding proteins, including the Arp2/3 complex, profilin, capping protein, and ADF/cofilins.

One of the dynamic processes in the cell that is controlled by F-actin assembly and disassembly is the lamellipodium. The lamellipodium of motile cell is predominantly composed of actin filaments, meaning that regulation of actin filament arrangement at the leading edge is necessary for the cellular directional motility [5].

ADF/cofilins are ubiquitous among eukaryotes and are essential proteins responsible for the turnover and reorganization of actin filaments *in vivo *[6, 7]. Mammals express three members of the ADF/cofilin (AC) family: actin depolymerising factor (ADF), nonmuscle cofilin (cofilin-1, Cfl1), and muscle cofilin (cofilin-2, Cfl2). Cofilin-1 is expressed in most cell types during development [8]. Cofilin-2 has two splice variants (Cfl2a and Cfl2b); Cfl2b is expressed; predominantly in muscle cells, while Cfl2a in several tissues [9]. Expression of ADF is restricted to endothelia and epithelia [8]. These three isoforms share similar but not identical biochemical activities. Only cofilin-1 and ADF are expressed in cultured human and mouse podocytes, with cofilin-1 being the predominant isoform [10, 11].

In this paper, we focus on the regulatory mechanisms of ADF/cofilin proteins in the modulation of actin dynamics in podocytes. We discuss how alterations in these processes can lead to a common phenotype in large variety of human glomerular diseases due to rearrangement of actin filaments in podocyte foot processes.

## 2. Modulation of Actin Dynamics by ADF/Cofilin

Actin filaments are a highly dynamic part of the cell which undergoes constant assembly and disassembly. Actin filaments are polymers that are composed of globular actin subunits. Each subunit is structurally polar and arranged head to tail to give the filament an overall structural polarity [12]. ADF/cofilin proteins modulate the actin dynamics during the mechanism of treadmilling.

Treadmilling is the dynamic process in which the overall length of the filaments remains approximately constant, but moves through growing at one end (plus, fast growing, or barbed end) by association of ATP-actin subunits (ATP-G-actin) and shrinking at the other end (minus, slow growing, or pointed end) through disassociation of monomers by ATP hydrolysis. When incorporated ATP-actin monomers undergo hydrolysis of ATP to form ADP-actin; cofilin, which displays greater affinity for ADP-actin, binds to ADP-actin which dissociates from the actin filament and recycles back to the monomer pool. ADF/cofilin proteins have the ability to enhance the rate of ADP-actin disassembly from the pointed end, which can be observed in the leading edge of motile cells [13]. Moreover, binding of ADF/cofilins to ADP actin filament destabilizes a twisted form of the actin filament [14, 15] and promotes severing of the filaments into short segments which increases the number of depolymerizing ends [16]. ADF/cofilin molecules binding to ADP-actin monomer can also inhibit nucleotide exchange to prevent its entrance to a new polymerization cycle [17].

On the other hand, ADF/cofilin proteins can accelerate spontaneous polymerization of monomers (nucleation) to initiate a new filament [18]. Nucleation of new filaments is dependent on ADF/cofilins concentration. At a low concentration, ADF/cofilin proteins have the highest-F-actin-severing activity but F-actin is stabilized and aged by ADF/cofilin decoration at higher concentration, and at a very high concentration, cofilin is able to nucleate new filaments [19]. This makes ADF/cofilin an important regulator not only of actin depolymerisation but also of actin stability and nucleation.

All three mammalian ADF/cofilin isoforms have a nuclear translocation sequence, perhaps enabling a ADF/cofilin-actin complex to pass into the nucleus [20]. The actin sequence lacks nuclear translocation signal but does have an export sequence [21]. With a molecular weight of 42 kDa, it is unlikely for actin to enter the nucleus by diffusion; therefore it relies on ADF/cofilin as transporter proteins to mediate its entry into the nucleus [21].

## 3. Regulation of ADF/Cofilins

In mammals phosphorylation of ADF/cofilin on Ser3 leads to inactivation [22, 23] but does not alter the protein conformation, while phosphorylation prevents G- and F-actin binding and tends to stabilize F-actin by inhibiting the ability of these protein to sever and depolymerize F-actin [24].

Phosphorylation of ADF/cofilins is mainly regulated by two kinase families, the LIM kinases (LIMK1, 2) [25, 26] and testicular protein kinases (TESK1, 2) [27, 28]. TESK expression is restricted to several tissues such as testis, brain, kidney, heart, and lung [29]. The most well-known pathways involved in the TESK activation are very different and mainly mediated by integrins and adhesion dependent [27, 28, 30]. LIM kinases are ubiquitously expressed and are downstream targets of small Rho-GTPases. Both LIMK1 and LIMK2, are targets of Rho-GTPases (Rho and Cdc42) via Rho kinases (ROCK1, ROCK2) and myotonic dystrophy kinase-related Cdc42-binding protein kinase (MRCK*α*), respectively [31, 32]. LIMK1, but not LIMK2 can be activated by p21-activated kinases (PAK1, PAK2, and PAK4), downstream of Rac and Cdc42 activation [33, 34]. In addition, LIMK1 can be activated in a Rho GTPase-independent manner [35, 36]. These evidences suggest that small Rho GTPases might regulate various actin-dependent cell functions through ADF/cofilin activity to maintain the structure and physiological function of adult kidneys.

**Figure 1:**
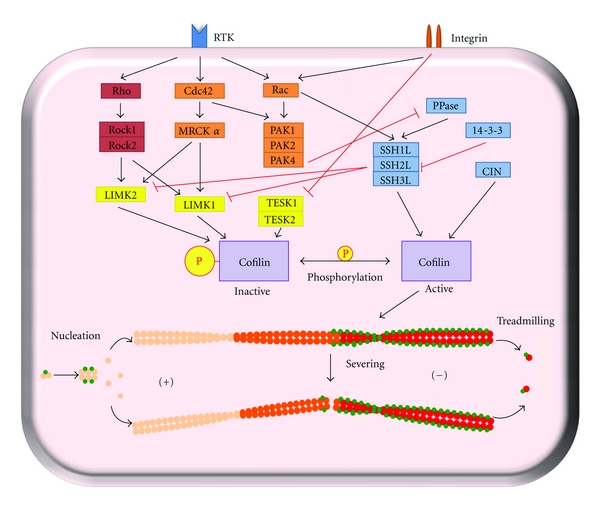
Regulatory pathways modulating cofilin phosphorylation and dephosphorylation Rho-GTPases are the predominant regulator of cofilin kinases and phosphatases. Cofilin phosphorylation is mainly regulated by LIMK and TESK. SSH family is the most important phosphatase that dephosphorylates cofilin directly or via LIMK inactivation. Phosphorylated cofilin can no longer bind and regulate the F-actin dynamics via treadmilling, severing, or nucleation.

ADF/cofilin can be dephosphorylated, and therefore activated, by two phosphatases, the slingshot family (SSH1L, SSH2L, and SSH3L) and chronophin (CIN) [37, 38]. CIN is highly specific for cofilin but the upstream signalling pathways remain a mystery [39]. SSH is the only known phosphatase to dephosphorylate and inactivate both LIMK1 and LIMK2 which leads to activation of ADF/cofilin by negative regulation of a negative regulator. Only a few regulatory pathways resulting in SSH activation have been ever identified. The phosphatase activity of SSH1L is negatively regulated via phosphorylation by PAK4 in different cell types [40]. It suggests a negative regulation of Rac1 activation on SSH and ADF/cofilin activity. In other cell types, SSH is activated through integrin pathway via Rac1 activation [41]. A colocalization of SSH and actin filaments together with the locatized activation of SSH1L was observed *in vitro*, indicating that assembly of F-actin can trigger the local activation of SSH1L and therefore promotes cofilin-mediated actin turnover in protrusive lamellipodia [42]. Some scaffolding proteins such as 14-3-3 can also participate in the modulation of ADF/cofilin-activity through interaction with SSH isoforms. Phosphorylation of SSH1L on serines 937 and 978 by protein kinase D (PKD) promotes the interaction of 14-3-3 with SSH1L and restricts its subcellular localization, which may inhibit SSH activity in breast carcinoma cells [42]. Furthermore, the activity of nonphosphorylated cofilin can be inhibited by binding to phosphatidylinositol 4,5-bisphosphate (PIP2), which prevents cofilin interaction with actin, but phospholipase-C- (PLC-) mediated PIP2 reduction causes cofilin to be released to cell membrane and to be activated [43]. As mentioned above, extracellular signals can regulate actin dynamics through ADF/cofilin and its upstream regulators (Figure 1).

 ADF/cofilin can also be mechanically controlled by intracellular pH both *in vivo* and *in vitro *[44, 45]. Changes in pH over the physiological range alter the severing capacity of active ADF/cofilin *in vitro*, but interestingly ADF is more sensitive to pH variation than cofilin [45]. Overall, the regulation of ADF/cofilin can be influenced by subcellular localization of ADF/cofilin kinases and phosphatases and synergistic-or competitive interactions of ADF/cofilins with other actin-binding proteins (ABPs) [46].

## 4. Regulation of ADF/Cofilin Activity in Podocytes

Experimental evidence indicates that nephrin, an Ig-G-like protein which is specifically expressed in podocytes is also engaged in regulating cofilin-1 and actin reorganization [11]. Garg et al. demonstrated that cofilin-1 colocalized at the plasma membrane with nephrin *in vitro*. Nephrin-induced activation of phosphatidylinositol 3 kinase (PI3K) is necessary for SSH1L dephosphorylation via an unknown phosphatase. SSH1L activation leads to cofilin-1 activation through LIMK dephosphorylation on Thr508 [40]. On the other hand, dephosphorylation of SSH1L decreases the affinity for 14-3-3, and the released SSH1L translocates to the protrusive leading edge of podocyte to activate cofilin-1- mediated actin remodelling. It still has to be clarified whether PKD is also involved in this regulation pathway in podocytes. Thus far no evidence was published that demonstrates that PKD can regulate SSH1L activity in podocytes.

Cofilin-1 activity can also be altered in response to several extracellular stimuli. Incubation of murine and human podocytes with TGF-*β*, a podocyte stressor, leads to increased cofilin-1 phosphorylation and decreased cofilin-1 activation [10]. In contrast, when stimulated with phorbol 12-myristate 13-acetate (PMA), increased activation of cofilin-1 was observed in murine and human podocytes. PMA activates PKC, a well-known regulator of actin cytoskeleton dynamics in large variety of cells [10]. Taken together, this suggests that PKC may be also involved in the pathway of modulating cofilin-1 activity. In human neutrophils, a PKC-dependent phosphorylation of cofilin was observed, but the involved PKC isoforms and the regulatory pathway remain to be demonstrated [47].

Experiments *in vitro* have proved that mechanical stress can change the podocyte morphology and the actin organization [48]. Osmotic stress, a major mechanical stress, has also been addressed to the cofilin-related regulation. In kidney tubular cells, hyperosmotic stress induces cofilin phosphorylation via Rho/ROCK/LIMK pathway and slightly delays actin kinetics due to reduced cofilin activation [49]. This same pathway was also activated by high-glucose treatment in cultured proximal tubular epithelial cells (PTECs), resulting in time-dependent increases in p-cofilin and pLIMK. Moreover, high glucose induced membrane translocation of Rho and ROCK2, without altering the PI3K-pathway, SSH1L, Rac/PAK, LIMK expression, or cofilin and SSH1L regulation at both mRNA and protein levels [50]. These studies highlight the possibility that osmotic stress or high glucose level may play a regulatory role in podocyte actin cytoskeleton through altering cofilin phosphorylation.

The motility and migration of podocytes can therefore be dramatically altered, when the expression level or activities of kinases or phosphatases that regulate ADF/cofilin is varied.

## 5. Podocyte Injury Associated with ADF/Cofilin Inactivation

The podocyte foot process contains a coordinated network of actin filaments which are connected by a multiprotein complex to the slit diaphragm and the glomerular basement membrane (GBM) via adhesion proteins. Proteins regulating or stabilizing the actin cytoskeleton are therefore essential for the maintenance of glomerular filtration function [51–53]. Rearrangement of the actin cytoskeleton and dysregulation of its associated proteins is the major cause of foot process effacement and proteinuria [54]. Foot process effacement can be observed in a variety of human and experimental glomerular diseases associated with massive proteinuria, including minimal change disease, focal segmental glomerulosclerosis (FSGS), membranous glomerulopathy, IgA-nephropathy, diabetic nephropathy, and lupus nephritis [55, 56]. Mutation of actin-binding proteins including *α*-actinin-4, MYH9, INF2, and CD2AP in podocytes leads to rearrangement of actin cytoskeleton, disruption of filtration barrier, and subsequent kidney failure [57–61].

There is ever-increasing evidence indicating that ADF/cofilins are the major regulators participating in actin turnover and cytoskeletal reorganization to sustain an intact podocyte foot processes. Different animal vertebrate models like knockdown or mutation of cofilin-1 in zebrafish or podocyte-specific knockout in mice have been performed to confirm the affect of cofilin-1 deficiency *in vivo*. Deficiency of cofilin-1 in zebrafish leads to a severe edematous phenotype, effacement of podocyte foot processes, and dysfunction of the glomerular filtration barrier indicating that cofilin-1 is an indispensable factor for the integrity of normal podocyte foot processes in zebrafish [10].

Mutant mice with podocyte-specific cofilin-1 deletion show disruption of renal function and alteration in podocytes foot processes at 6 months of age. The mutant mice have severe proteinuria and indiscernible foot process spreading. However podocyte foot processes remain intact in newborn mutant mice [11]. The delayed set-on of proteinuria and podocyte foot process effacement can be explained by compensation of increased ADF isoform expression during the early development of mutant mice. Some studies revealed that the different isoforms of ADF/cofilin are not completely redundant. ADF is efficient at turning over actinafilaments, whereas cofilin-1 is a more effective nucleator of new filament assembly [8, 62, 63]. However, due to the differences in actin modulation functions, ADF can not completely compensate the lost function of cofilin-1 and it does not stay continuously upregulated in the podocytes-specific knockout mouse. Interestingly, proteinuria and phenotypic changes coincide with downregulated ADF expression [11].

*In vitro* cofilin-1 deficiency does not lead to significant changes in actin architecture in podocytes. Supression of cofilin-1 expression in cultured podocytes resulted in a limited breakdown and formation of new actin filaments beneath the plasma membrane and loss of forward pressure on the overlying membrane, which leads to a reduced cellular migration activity, suggesting that cofilin-1 activity is required for rapid actin turnover in the lamellipodial protrusion and is necessary for directional cellular migration activity in podocytes [10, 11]. However, regulation of actin dynamics is not the only role of actin to maintain the podocyte function. Obrdlik and Percipalle. showed that cofilin-1 is required for elongation of RNA polymerase-II-mediated transcription through interaction with actin [64]. This study indirectly indicates that deletion or downregulation of cofilin-1 might disrupt the transcription of nascent genes that are essential for podocyte integrity.

Despite wide distribution of cofilin modulators genes, deletion of these genes resulted in relatively mild phenotypes in mice. Deficiency of LIMK-1 led to abnormalities in synaptic structure and spine development, due to aberrant regulation of the actin cytoskeleton *in vivo* [65]. LIMK-2 knockout mice exhibited minimal abnormalities, while the double LIMK-1/LIMK-2 null mice were more severely impaired but not embryonic lethal [66]. These morphological and functional changes were primarily observed in the neuronal system, but still suggest the possibility that LIMK deficiency might cause similar abnormalities in podocyte structure and function. SSH3L knockout mice were made to examine its potential roles *in vivo*. Unexpectedly SSH3L was not essential for viability or development of epithelial tissues [67]. An SSH1L or SSH2L deficiency in animal models or human diseases was not yet reported.

Under pathological conditions in the kidney, alterations of the extracellular milieu also change cofilin-1 activity. TGF-*β* is described as a causative factor for initiation and progression of proteinuric diseases in mice and humans. TGF-*β* accumulates in injured kidneys in experimental animal models and chronic renal disease in humans [68, 69]. In different disease states TGF-*β* activation induces a constant cofilin-1 inactivation, which results in disruption of cofilin-1-mediated actin dynamins and subsequently effacement of podocytes and proteinuria. TGF-*β* is an important mediator of progressive fibrosis, cell proliferation, and cell death in glomerular diseases. TGF-*β* pathways also occupy a central position in signalling networks that control a diverse set of cellular processes. Interestingly, the effects of TGF-*β* on podocytes are concentration dependent [70]. Our group is currently investigating whether TGF-*β* impairs directional migration activity and leads to alternations in cytoskeleton arrangement in a concentration-dependent manner in podocytes. As mentioned above, PMA and TGF-*β* have opposite effect on cofilin phosphorylation. PMA has already been shown to increase migration in several cell types [71, 72]. However, it is still unknown whether PMA can rescue the dysfunction of TGF-*β* induced in podocytes.

Hyperglycemia is a prerequisite for development of diabetic nephropathty. Hyperglycemia induces increased osmolarity of blood serum. In diabetic mellitus the high glucose level and hyperosmolarity could promote the Rho/ROCK activation in podocytes, because abundant evidence identified high glucose and osmotic stress as stimulators of Rho-ROCK signalling pathway [73–76]. It suggests that Rho activation can cause cofilin phosphorylation and inactivation in podocytes. The disruption of actin dynamic via cofilin inactivation dependent on the hyperglycemia and hyperosmotic stress is one of the causative stimulators for the progressive development of diabetic nephropathy. In addition, high glucose level leads to increased expression of TGF-*β* [77], which further enhances the cofilin inactivation and its nuclear localization. In response to the stimulation by TGF-*β*, phosphorylated cofilin-1 undergoes nuclear translocation in both murine and human podocytes (our unpublished data).

**Figure 2:**
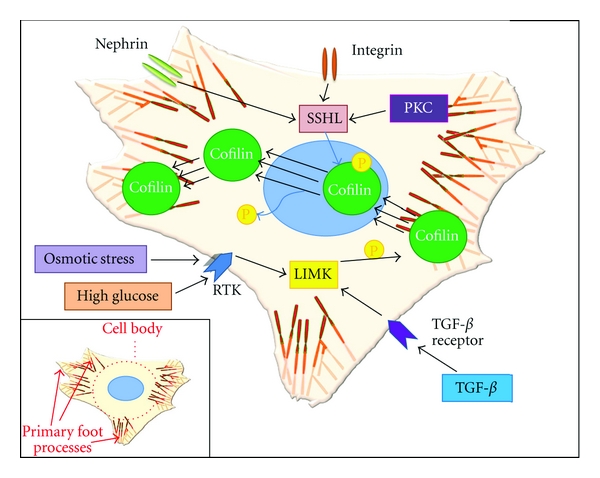
Central role of cofilin in podocyte effacement TGF-*β* or high-glucose stimulation triggers cofilin-1 phosphorylation. Phosphorylated/inactivated cofilin-1 undergoes translocation from cytoplasma to nucleus and is therefore not able to bind and promote F-actin rearrangement. Nephrin and integrin cluster or PKC activate the cofilin-1 via SSH1L activation. A rapid turnover of cofilin-1 is essential for the actin cytoskeleton dynamics in podocyte to perpetuate podocyte integrity. Secondary foot processes of podocyte are not shown here. Secondary foot processes are fine actin-rich processes that sprout out of primary processes and interdigitate with foot processes of neighbouring podocytes.

In renal diseases associated with foot process effacement, cofilin-1 was inactivated and translocated to the nucleus of podocytes. Cofilin-1 is dephosphorylated and active under normal homeostasis conditions. In contrast, when the podocytes undergo foot process effacement because of nephritic glomerular diseases, cofilin-1 was found phosphorylated (inactivated) and translocated to the nucleus of podocytes [10] (Figure 2). This suggested that cofilin-1 can be a potential diagnostic marker to detect the injury of podocytes in the glomerulus. The role of nuclear uptake of phosphorylated cofilin is currently unknown. But some evidence indicates that cofilin forms a complex with actin and DNaseI and perhaps plays a role in DNA degradation and initiation of apoptosis [78]. One of the new most interesting findings is that even though cofilin-1 is constantly expressed throughout podocytes, the phosphorylated form is not detectable in the normal glomerulus in mice and humans indicating that all of the cofilins are active. Only if proteinuria is present, there is a dramatic increase in phosphorylated cofilin-1 and nuclear translocation of phosphocofilin is detectable [10]. The expression of phosphorylated cofilin-1 in glomerular diseases suggests a reduced capacity of podocytes to adapt to glomerular pressure differences. A higher filtration pressure and distension of the capillary wall can not be compensated and leads to proteinuria.

Because the glomerular capillary pressure constantly changes with blood pressure, it is likely that the foot processes experience distension of the capillary wall. Therefore, podocytes must be able to adapt to these changes to assure a network of functional filtration slits. Cofilin-1 is therefore necessary for foot process spreading by accelerating actin turnover and gives the pushing force for the protrusive leading edge. When podocytes are injured, cofilin-1 is required to restore the normal actin architecture of podocytes for recovery. Otherwise, this injury is not reversible and results in renal diseases associated with podocytes effacement and massive proteinuria. Thus, cofilin dephosphorylation might be an attractive pharmacological target to ensure proper actin turnover in proteinuric diseases which might help in the recovery process of effacement.
